# Preferential Identification of Agonistic OX40 Antibodies by Using Cell Lysate to Pan Natively Paired, Humanized Mouse-Derived Yeast Surface Display Libraries

**DOI:** 10.3390/antib8010017

**Published:** 2019-02-19

**Authors:** Angélica V. Medina-Cucurella, Rena A. Mizrahi, Michael A. Asensio, Robert C. Edgar, Jackson Leong, Renee Leong, Yoong Wearn Lim, Ayla Nelson, Ariel R. Niedecken, Jan Fredrik Simons, Matthew J. Spindler, Kacy Stadtmiller, Nicholas Wayham, Adam S. Adler, David S. Johnson

**Affiliations:** 1 Department of Chemical Engineering and Material Science, Michigan State University, East Lansing, MI 48824, USA; medinacu@msu.edu; 2 GigaGen Inc., One Tower Place, Suite 750, South San Francisco, CA 94080, USA; rmizrahi@gigagen.com (R.A.M.); masensio@gigagen.com (M.A.A.); robert@drive5.com (R.C.E.); jleong@gigagen.com (J.L.); rleong@gigagen.com (R.L.); ylim@gigagen.com (Y.W.L.); anelson@gigagen.com (A.N.); aniedecken@gigagen.com (A.R.N.); jsimons@gigagen.com (J.F.S.); mspindler@gigagen.com (M.J.S.); kstadtmiller@gigagen.com (K.S.); nwayham@gigagen.com (N.W.); aadler@gigagen.com (A.S.A.)

**Keywords:** OX40, humanized mouse antibody repertoires, deep sequencing, yeast display

## Abstract

To discover therapeutically relevant antibody candidates, many groups use mouse immunization followed by hybridoma generation or B cell screening. One modern approach is to screen B cells by generating natively paired single chain variable fragment (scFv) display libraries in yeast. Such methods typically rely on soluble antigens for scFv library screening. However, many therapeutically relevant cell-surface targets are difficult to express in a soluble protein format, complicating discovery. In this study, we developed methods to screen humanized mouse-derived yeast scFv libraries using recombinant OX40 protein in cell lysate. We used deep sequencing to compare screening with cell lysate to screening with soluble OX40 protein, in the context of mouse immunizations using either soluble OX40 or OX40-expressing cells and OX40-encoding DNA vector. We found that all tested methods produce a unique diversity of scFv binders. However, when we reformatted forty-one of these scFv as full-length monoclonal antibodies (mAbs), we observed that mAbs identified using soluble antigen immunization with cell lysate sorting always bound cell surface OX40, whereas other methods had significant false positive rates. Antibodies identified using soluble antigen immunization and cell lysate sorting were also significantly more likely to activate OX40 in a cellular assay. Our data suggest that sorting with OX40 protein in cell lysate is more likely than other methods to retain the epitopes required for antibody-mediated OX40 agonism.

## 1. Introduction

Many antibody drugs bind to disease targets expressed on cell surfaces. For example, antibodies may bind to the surface of tumor cells and induce antibody-dependent cellular cytotoxicity (ADCC). Conventionally, antibody drug discovery groups use either hybridomas [1] or phage display [2] to discover antibody drugs. Hybridomas are typically screened for cell surface binders using enzyme-linked immunosorbent assays (ELISAs) in 96-well plates [3]. Hybridoma methods, therefore, require expensive robotics to screen thousands of antibody candidates. Phage display has a much higher throughput, because billions-diverse phage libraries can be panned against cells affixed to well plates [4]. However, most therapeutic antibodies have been discovered in mice [5], perhaps due to difficulties with developability of artificial antibodies, such as low solubility binders discovered in phage display [6,7].

Recently, we invented a novel method for screening millions-diverse antibody repertoires using microfluidics, yeast display, and deep sequencing [8,9,10]. Our method leverages the developability advantages of naturally paired antibodies with the massively parallel throughput of display technologies. Other groups later further validated our work with similar methods [11,12]. However, our previously published methods required soluble antigen for both mouse immunization and fluorescence-activated cell sorting (FACS). This limitation excluded the possibility of using the method to identify antibodies against multi-pass transmembrane proteins, such as G-protein coupled receptors. Additionally, the requirement for soluble protein may lead to antibodies directed against spurious epitopes not present in the native conformation on the surface of target cells.

OX40, or tumor necrosis factor receptor superfamily member 4 (TNFRSF4), is a costimulatory immune receptor transiently expressed on T cells which upregulates T cell activity upon binding to its ligand, OX40L. Therapeutic agonism of OX40 may increase T cell differentiation and tumor killing functions [13]. Agonism requires a ligand binding to OX40 in a way that generates complexes of crosslinked OX40 molecules on cell surfaces [14]. Although the crystal structure of OX40 binding to OX40L has been resolved [15], the specific epitopes required for agonism are not well understood. Development of novel therapeutic antibodies would benefit from a method that generates large panels of antibodies directed against a variety of OX40 epitopes that are bioavailable at the cell surface.

To improve OX40 antibody discovery, we adapted our previously published methods [8,9,10] to test different immunization methods (cells versus soluble antigen) and different antibody selection methods (cell lysate versus soluble antigen). The cell lysate selection method was adapted from prior work [16,17], specifically by using a peptide tag rather than biotin to label the cell lysate. We synthesized forty-one monoclonal antibodies (mAbs) from the various methods and found that soluble OX40 soluble antigen immunization followed by sorting with cell lysate was most likely to identify antibodies that bind cell surface antigen and yielded more antibodies that activate OX40 in cellular assays.

## 2. Materials and Methods

### 2.1. Mouse Immunization and Sample Preparation

All mouse work was performed at Antibody Solutions (Sunnyvale, CA, USA) and overseen by a licensed veterinarian. All experiments were performed using mice from Trianni (San Francisco, CA, USA), which are C57BL/6 that transgenically express a complete repertoire of fully human immunoglobulin gamma (IgG) and immunoglobulin kappa (IgK) V(D)J genes, but retain mouse promoters, introns, and constant domains.

For the soluble OX40 immunizations, five Trianni mice were immunized with recombinant His-tagged human OX40 extracellular domain (Acro OX40-H5224, Newark, DE, USA), using ALD/MDP (alhydrogel/muramyl dipeptide) as an adjuvant. 10 μg of OX40 protein with adjuvant was injected into the footpad twice per week for three weeks. We assessed titer at Day 21 with ELISA, using a dilution series of antigen, ranging from 1000 ng/mL to 1 ng/mL and goat anti-mouse IgG-HRP (Jackson ImmunoResearch 115-035-071, West Grove, PA, USA) (Supplementary Figure S1). After assessing serum titer, two more footpad boosts of 10 μg without adjuvant were administered to each animal before sacrifice.

For the cells/DNA OX40 immunizations, we first transfected Flp-In 3T3 cells (Thermo Fisher Scientific, Waltham, MA, USA) with a vector encoding un-tagged, full-length human OX40 (Supplementary Figure S2). A pool of OX40-positive cells was selected using Hygromycin B (Gemini Bio 400123, West Sacramento, CA, USA) for 2 weeks. Cells were treated with Mitomycin C before cryopreservation. One to two million cells were injected per mouse. A footpad injection was performed with three Trianni mice on day 0 with cells, then days 3, 7, and 10 with 20 µg DNA plasmid encoding full-length, untagged human OX40, then day 14 with cells, day 17 and 21 with DNA, and final boosts on Days 24 and 27 with cells prior to tissue harvest. Before the final boosts, mouse serum titer was assessed with flow cytometry, using a dilution series of each animal’s serum, starting at 1:200 and ending at 1:145,000 (Supplementary Figure S3). Briefly, the same 3T3 cells stably expressing OX40 were incubated with the serum dilution, washed, and then stained with goat anti-mouse IgG-rPE (Jackson ImmunoResearch, 115-116-071, West Grove, PA, USA). The final library was generated from two of the three mice, as the third mouse died prior to tissue harvest.

We surgically removed lymph nodes (popliteal, inguinal, axillary, and mesenteric) and spleens from the sacrificed animals. Single cell suspensions for spleen and lymph nodes were made by manual disruption followed by passage through a 70 μm filter. We used the EasySep™ Mouse Pan-B Cell Isolation (Stemcell Technologies, Vancouver, Canada) negative selection kit to isolate B cells from the single cell suspensions. Cells were stained for viability using Trypan blue and then quantified with a C-Chip hemocytometer (Incyto, Chungnam-do, Korea). We then diluted the cells to 6000 cells/μL in PBS with 12% OptiPrep™ Density Gradient Medium (Sigma, St. Louis, MO, USA). The purified cell populations were used for microfluidic encapsulation as described below.

### 2.2. Generating Paired Heavy and Light Chain Libraries

As described previously [8,9,10], the generation of libraries comprised of three steps: (i) poly(A)+ mRNA capture, (ii) multiplexed overlap extension reverse transcriptase polymerase chain reaction (OE-RT-PCR), and (iii) nested PCR to remove artifacts and add adapter sequences for deep sequencing or yeast display libraries.

Briefly, we isolated 1.6–1.9 million B cells into fluorocarbon oil (Dolomite, Royston, UK) emulsion microdroplets (Supplementary Table S1) with a lysis buffer (20 mM Tris pH 7.5, 0.5 M NaCl, 1 mM EDTA, 0.5% Tween-20, and 20 mM DTT) and oligo(dT) beads (New England BioLabs, Ipswich, MA, USA), using an emulsion droplet microfluidic chip [8,9,10]. We purified beads from the droplets using Pico-Break solution (Dolomite, Royston, UK).

We then performed multiplex OE-RT-PCR in emulsions, using purified RNA-bound beads as a template, as described elsewhere [8,9,10]. The OE-RT-PCR product was gel purified and PCR was performed to add adapters for Illumina sequencing or yeast display; for sequencing, a randomer of seven nucleotides was added to increase base calling accuracy in subsequent next generation sequencing steps. Nested PCR is performed with 2× NEBNext High-Fidelity amplification mix (New England BioLabs, Ipswich, MA, USA) with either Illumina adapter containing primers or primers for cloning into the yeast expression vector.

### 2.3. Yeast Library Screening

*Saccharomyces cerevisiae* EBY100 cells (ATCC, Manassass, VA, USA) were electroporated (Bio-Rad Gene Pulser II; 0.54 kV, 25 uF, resistance set to infinity) with gel-purified nested PCR product and linearized pYD vector [8,9,10] for homologous recombination in vivo. Transformed cells were expanded and induced with galactose to generate yeast scFv display libraries.

For the soluble OX40 FACS experiments, human OX40-His (described above) protein was biotinylated using the EZ-Link Micro Sulfo-NHS-LC-Biotinylation kit (Thermo Fisher Scientific, Waltham, MA, USA). The biotinylation reagent was resuspended to 9 mM and added to the protein at a 50-fold molar excess. The reaction was incubated on ice for 2 h, and then the biotinylation reagent was removed using Zeba desalting columns (Thermo Fisher Scientific, Waltham, MA, USA). The final protein concentration was calculated with a Bradford assay. The scFv libraries were then stained with anti-c-Myc (Thermo Fisher Scientific A21281, Waltham, MA, USA) and an AF488-conjugated secondary antibody (Thermo Fisher Scientific A11039). Biotinylated OX40 was added to the yeast culture (250 nM final concentration) and stained with APC-streptavidin (Thermo Fisher Scientific, Waltham, MA, USA). Approximately two million cells were then flow sorted on a FACSMelody (BD, San Jose, CA, USA) for double positive cells (AF488+/APC+). Populations of binder scFv clones were recovered, expanded, and then subjected to a second and third round of FACS with the same antigen at 250 nM final concentration. A fourth round of FACS was additionally performed on select samples (Supplementary Figure S4).

For the cells/DNA OX40 FACS experiments, we engineered an expression vector that expresses full-length human OX40 fused to a FLAG peptide at the N-terminus (Supplementary Figure S2). This vector was used to stably transfect CHO cells via targeted genome integration. Approximately 12.5 × 10^6^ OX40-positive transfected cells encoding full-length human OX40 were used to prepare the cell lysate for each staining condition. First, cells were harvested and washed twice with 10 mL of ice-cold PBS. Second, cells were resuspended in a lysis buffer (PBS, 1% Triton X-100, 2 mM EDTA, and 1 × protease inhibitor cocktail) to a final concentration of 5 × 10^7^ cells/mL and were incubated, rotating for 30 min at 4 °C [16]. Finally, cells were harvested and the supernatant (the detergent-solubilized cell lysate) was removed to a fresh tube and stored at 4 °C until use. The final total protein concentration in the lysate was calculated using a Bradford assay. The scFv yeast libraries were labeled with 250 µL of cell lysate and incubated, rotating, overnight at 4 °C. The next day, labeled yeast cells were stained with anti-c-Myc, an AF488-conjugated secondary antibody, and APC anti-FLAG (clone L5, BioLegend 637308, San Diego, CA, USA). Approximately, four million cells were flow sorted on a FACSMelody. As described above, the collected populations of binder scFv clones were recovered, expanded, and subjected to two additional rounds of FACS using the same cell lysate concentration.

### 2.4. Sequence Analysis

Libraries were sequenced on a MiSeq (Illumina, San Diego, CA, USA) using a 500 cycle MiSeq Reagent Kit v2, as described previously [8,9,10]. Sequencing was performed in two different runs. In the first run, we directly sequenced the scFv libraries to obtain a forward read of 357 cycles for the light chain complementarity-determining region (CDR)3 and V-gene, and a reverse sequence read of 162 cycles across the heavy chain CDR3 and part of the heavy chain V-gene. In the second run, we first used the scFv library as a template for PCR to independently amplify heavy and light chain V-genes. We then obtained a forward read of 255 cycles and a reverse read of 255 cycles for the heavy and light chain Ig separately. The second run yields overlapping reads, which is useful for sequencing error correction.

We used previously published methods for error correction, reading frame identification, and FR/CDR junction calls [8,9,10,18]. We discard reads with E > 1 (E is the expected number of errors), retaining sequences for which the most probable number of base call errors is zero. We also discard singleton nucleotide reads to further improve confidence in antibody sequences. In order to identify V and J gene families and calculate percent identity to germline, we aligned antibody nucleotide sequences with the IMGT database [19].

We define “clones” conservatively, with an emphasis on sequence accuracy. First, we concatenated the CDR3K and CDR3H amino acid sequences from each scFv sequence into a single contiguous amino acid sequence. Next, we used USEARCH [20] to compute the total number of amino acid differences in all pairwise alignments between each concatenated sequence in each data set. Groups of sequences with ≤2 amino acid differences in the concatenated CDR3s were counted as a single clone. Finally, we used the majority amino acid identity at each residue position to generate the consensus amino acid sequence of the clone from sequences of the members of the group.

To generate clonal cluster plots, we first used USEARCH [20] to generate all pairwise alignments across the complete set of FACS-sorted IgH and IgK scFv sequences (Supplementary Tables S2–S9). We then computed the total number of amino acid differences between each scFv sequence. We then generated clustering plots using the igraph R package [21], using the “layout_with_graphopt” option. Antibody clones are represented by “nodes” in the plots. The size of the nodes corresponds to the frequency of the antibody clone in the FACS-sorted population: small (<2% frequency), medium (2–12% frequency), and large (>12% frequency). An “edge” (a line linking nodes) was drawn between any sequences with ≤9 amino acid differences in the concatenated CDR3s.

### 2.5. Monoclonal Antibody Expression and Characterization

We synthesized mAbs by cloning antibody sequences into a variant of the pCDNA5/FRT mammalian expression vector (Thermo Fisher Scientific, Waltham, MA, USA), as described previously [8,9,10]. Expression constructs were prepared using a BioXP™ robotic workstation (SGI DNA, La Jolla, CA, USA). Human IgHG1 isotype was used for all constant domains. MAb plasmids were then transiently transfection into ExpiCHO cells (Thermo Fisher Scientific, Waltham, MA, USA). Transfected cells were cultured in ExpiCHO medium for 7–9 days. An IgG ELISA kit (Abcam, Cambridge, UK) was used to quantify the concentration of antibody in the supernatants.

To measure cell surface binding, we first generated stable human OX40-expressing Flp-In Chinese hamster ovary (CHO) cells (Thermo Fisher Scientific, Waltham, MA, USA). One million cells (1:1 mix of OX40 and irrelevant PD-1-expressing negative control cells) were stained with 1 μg of anti-OX40 mAb in 100 μL MACS Buffer (DPBS with 0.5% BSA and 2 mM EDTA) for 30 min at 4 °C. Cells were then co-stained with anti-human IgG Fc-PE (BioLegend clone M1310G05, San Diego, CA, USA) and anti-human PD-1-APC (BioLegend clone EH12.2H7, San Diego, CA, USA) antibodies for 30 min at 4 °C. We then used a FACSMelody (BD, San Jose, CA, USA) quantify binding. We used FlowJo to determine the intensity of the OX40-expressing cells versus the irrelevant negative controls (Supplementary Figure S5).

For measurement of the kinetics of binding to soluble OX40, 5 μg/mL antibodies were loaded onto a Protein A biosensor using the Octet Red96 system (ForteBio, Fremont, CA, USA) by a contract research organization (Bionova, Fremont, CA, USA). Loaded biosensors were dipped into His-tagged OX40 extracellular domain (Acro OX40-H5224, Newark, DE, USA) at 200 nM, 100 nM, and 50 nM, or 1600 nM, 800 nM, and 400 nM, depending on the strength of the response to the OX40 antigen binding the mAb. Kinetic analysis was performed using a 1:1 binding model and global fitting (Supplementary Figure S6).

To determine the ability of each mAb to activate OX40 in vitro, we used a kit (Promega, Madison, WI, USA) according to the manufacturer’s instructions. We performed OX40 activation assay in the presence of cells expressing FcγRIIB, which simulates the putative in vivo mechanism of OX40 cross-linking [14]. On the day prior to the assay, FcγRIIB/CHO-K1 cells were thawed into 95% RPMI 1640/5% FBS and plated into 96-well plates. After incubating for 5–7 h at 37 °C, 5% CO_2_, OX40-expressing Jurkat cells were thawed and added to the wells containing FcγRIIB/CHO-K1 cells. After incubating the cell mixtures overnight, antibodies were diluted in 95% RPMI 1640/5% FBS. The antibody dilutions were then added to the wells containing the cells. The cell/antibody mixtures were incubated at 37 °C, 5% CO_2_ for 5 h, after which we added Bio-Glo Reagent. Luminescence was read using a Spectramax i3x plate reader (Molecular Devices, San Jose, CA, USA). IC50 was calculated by plotting RLU (relative luminescence units) vs concentration using SoftMax Pro (Molecular Devices, San Jose, CA, USA) (Supplementary Figure S7). In-house produced pogalizumab was used as a positive control, and an antibody binding to an irrelevant antigen was used as a negative control.

## 3. Results

### 3.1. Overview of the Experimental Approach

First, we stably expressed full-length human OX40 protein in mouse 3T3 cells. Next, we immunized transgenic humanized Trianni mice with either OX40-expressing 3T3 cells or soluble OX40 extracellular domain using a rapid immunization protocol. The cohort of mice immunized with OX40-expressing 3T3 cells was additionally boosted with a DNA vector driving expression of full-length OX40 protein. All mice were checked for anti-OX40 serum titer (Supplementary Figures S1 and S3) and sacrificed after approximately four weeks. Spleen and lymph nodes were disaggregated into single cell solutions, tissues from replicate animals were pooled, and B cells were isolated from the single cell solutions and cryopreserved.

**Figure 1:**
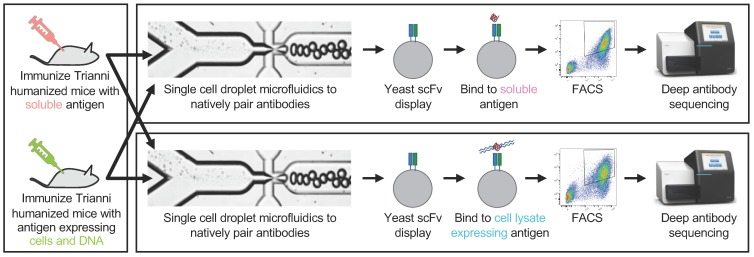
Overview of the generation and screening of scFv libraries derivates from B cells from humanized mice with either soluble OX40 or cells and DNA expressing OX40. B cells are isolated from spleen and lymph nodes. Next, B cells are encapsulated into droplets with oligo-dT beads and a lysis solution to generate DNA amplicon that encodes the scFv libraries with native pairing heavy and light Ig. The scFv libraries are then transfected into yeast cells and labeled with either soluble OX40 or lysate from cells expressing OX40. Next, FACS is used to collect scFv with the highest FACS signal. Finally, deep sequencing is used to identify all clones in the pre- and post-sort populations.

We then used droplet microfluidics [8,9,10] to isolate millions of single cells from each experimental arm (Supplementary Table S1) into aqueous-in-oil picoliter droplets. Cells were lysed inside the droplets, and mRNA from the single cells was bound to oligo(dT) beads. The oligo(dT) beads were then injected into a second emulsion with multiplex primers that amplify heavy and light chain Ig. The primers are designed with overlapping linker sequences that physically link heavy and light chain Ig into scFv expression constructs. The linked Ig libraries are subjected to deep sequencing to quantify clonal antibody diversity (Supplementary Table S1). Each library was then electroporated into yeast for scFv display (Figure 1).

**Figure 2:**
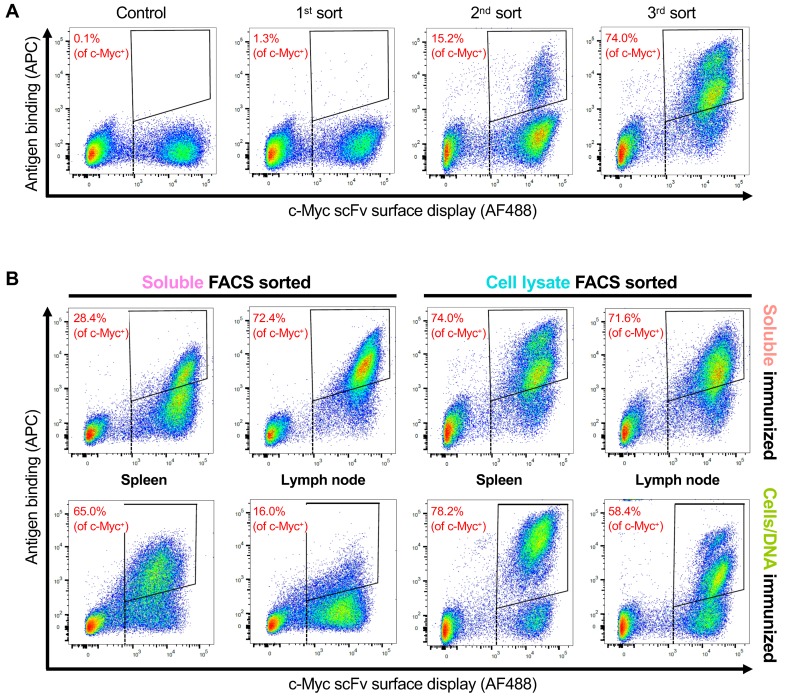
scFv libraries from immunized mice subjected to FACS selection for OX40. An anti-c-Myc (AF488) staining is used to identify yeast displaying scFv on the cell surface (*x*-axis). APC-streptavidin is used to identify yeast cells with biotinylated soluble OX40 bound, and anti-FLAG (APC) staining is used to identify yeast cells bound to lysate from cells expressing OX40-FLAG (*y*-axis). A negative control is used to set a quadrangle gate for the FACS selection (upper right corner). The percentage in each quadrangle represents the proportion of c-Myc positive yeast cells that fell within the gate. (**A**) An example of the soluble immunized, cell lysate sorted, spleen library subjected to three rounds of sorting to enrich the cells for positive antigen binding and scFv display on yeast surface. (**B**) FACS plots showing the percentage of enriched antibodies after the three rounds of sorting under each condition. Note that the image used for soluble immunized, cell lysate sorted, spleen is the same image as the 3rd sort from A.

Next, each yeast scFv library was subjected to FACS using either lysate from FLAG-tagged OX40-expressing CHO cells or soluble His-tagged OX40 extracellular domain (Figure 2). Cell lysate is prepared by lysing recombinant cells in a buffer containing a surfactant (1% Triton X-100) and quantified using a Bradford assay. The cell lysate was then incubated with each yeast scFv library, stained with an anti-FLAG secondary antibody and anti-c-Myc staining to quantify expression of scFv, and then subjected to FACS to pan for antigen-positive, c-Myc-positive binders. FACS with soluble OX40 was performed as described previously [8,9,10]. We performed either three or four rounds of FACS panning and deep sequenced the binders.

Finally, to compare the functional characteristics of antibodies identified with each experimental method, we synthesized forty-one monoclonal antibodies from the panning experiments. We chose a sample of antibodies from distinct putative clonal lineages, with an emphasis on the most common clones from each experimental method (Supplementary Tables S2–S9). We then used the full-length mAbs to perform kinetics measurements, cell surface binding assays, and in vitro cellular activation assays.

### 3.2. Analysis of Serum Titers

Soluble OX40 antigen yielded consistently high anti-OX40 serum titers in five replicate mice (Supplementary Figure S1). We pooled the splenocytes or lymph nodes from these five animals to produce one yeast scFv library for each tissue type, for a total of two soluble immunization OX40 libraries. Immunizations using OX40 cells/DNA were less consistent across three replicate mice, generating a non-responder, a medium responder, and a high responder (Supplementary Figure S3). Splenocytes or lymph nodes from two of three animals were pooled to produce a single natively paired yeast scFv library for each tissue type (the medium responder animal died prior to tissue harvest). Thus, we generated a total of four natively paired yeast scFv libraries (2 tissues × 2 immunization methods = 4 libraries; Supplementary Table S1).

### 3.3. Selection of OX40 scFv Binders with FACS

Prior publications have described protocols for panning yeast surface scFv display libraries with biotinylated, detergent-solubilized cell lysate [16,17]. We reasoned that biotinylation was suboptimal because the method labels all proteins in the cell lysate rather than only the target protein, leading to a loss of specificity in sorting and additional labor-intensive steps for every panning experiment. Therefore, we developed an approach based on an OX40 protein fused to a FLAG peptide tag. Briefly, we engineered an expression vector that expresses full-length human OX40 fused to a FLAG peptide at the N-terminus (Supplementary Figure S2). This vector was used to stably transfect CHO cells via targeted genome integration. Fresh cell lysate was prepared for each panning experiment by lysing recombinant cells in a buffer containing a surfactant (1% Triton X-100).

In a typical experiment, we obtained around 7–8 mg/mL of total protein concentration per 5.0 × 10^7^ cells/mL. Prior work has shown that the sensitivity and specificity of scFv binder discovery are functions of the molarity of a soluble target used during panning [8,9,10]. We, therefore, tested panning with four different concentrations of cell lysate (Supplementary Figure S8). After two rounds of panning, the fraction of scFv binders was as low as 6.4% (0.475 mg/mL) and as high as 31% (3.8 mg/mL). The FACS plots at 1.9 mg/mL of cell lysate were qualitatively and quantitatively similar to our prior panning experiments using various soluble antigens at 7–70 nM [8,9,10]. Therefore, we used approximately 2 mg/mL of cell lysate for all subsequent cell lysate panning experiments.

Although all tissues, from both immunization methods, and with both FACS methods yielded scFv binders, there were qualitative and quantitative differences in the FACS plots (Figure 2). After three rounds of panning, the fraction of scFv binders was as low as 16.0% (cells/DNA immunization, soluble antigen FACS) and as high as 78.2% (cells/DNA immunization, cell lysate FACS). On average, the soluble immunogen yielded a higher fraction of scFv binders than the cells/DNA immunization (61.6% versus 54.4%, respectively), and the cell lysate FACS yielded a higher fraction of scFv binders than the soluble antigen FACS (70.6% versus 45.5%, respectively). Because the cells/DNA immunogen followed by soluble FACS yielded a lower fraction of scFv binders, we performed a fourth round of panning on these libraries (Supplementary Figure S3), which improved the fraction of scFv binders by as much as 58.3% (from 16.0% to 74.3%), suggesting an increase in specificity. In general, cell lysate FACS produced a more significant shift in FLAG-APC fluorescence (antigen binding) than soluble antigen FACS.

### 3.4. Sequence Characteristics of OX40 scFv Binders

We deep sequenced the yeast scFv libraries before and after FACS (Supplementary Table S1), as described previously [8,9,10]. Note that we use extremely conservative error processing, which favors clone sequence quality over capturing the “long tail” of clonal diversity. Before FACS, the scFv libraries contained between 16,491 and 19,509 clones. After FACS, the scFv libraries were much more oligoclonal, containing between 61 and 238 clones.

We analyzed the most common (≥0.1% frequency) scFv sequences to determine pre- versus post-FACS clonal enrichments achieved by each method. We did not observe statistically significant differences between the mean clone counts of soluble versus cells/DNA immunizations, or between soluble versus cell lysate sorts (*p* > 0.01, *t*-test). The average pre-sort scFv clone abundance was 0.032%, with a range from 0% (not detected) to 0.71%. Sequences present in the post-sort libraries were not detected in the pre-sort libraries for 54/268 (20.1%) of clones, suggesting that many candidate binder clones were extremely rare in the mouse repertoires. The average enrichment between pre- and post-sort clone counts was 5056-fold, with a range from 2.2-fold to 500,000-fold. We note that prior work on Balb/c, SJL, and Medarex HuMAb mice [9,10] yielded similar levels of enrichment and clonal diversity both before and after FACS.

**Figure 3:**
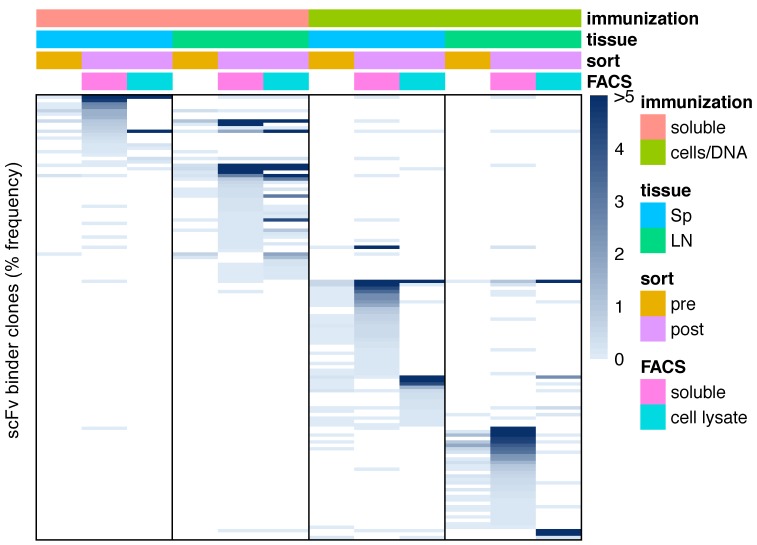
Overlapping clones in the pre- and post-sort populations obtained from each experimental parameter. Clone frequencies are represented by the blue heatmap, with unique clones aligned into rows across the repertoires. We only show clones that are present with frequencies of 0.1% or higher in at least one of the post-FACS repertoires. We organize the repertoires by pre- vs. post-FACS, tissue of origin (lymph nodes vs. spleen), immunization method (soluble antigen vs. cells/DNA), and panning condition (soluble antigen vs. cell lysate).

Next, to determine whether different methods discover the same scFv binders, we analyzed the most common (≥0.1% frequency) scFv sequences for overlap between each post-FACS library (Supplementary Tables S2–S9). Only 6.8% of enriched clones (16/235 non-redundant, unique clones) were shared between at least two of the eight series of scFv panning series (2 tissues × 2 FACS methods × 2 immunization methods) (Figure 3), suggesting different immunization methods and different FACS methods typically capture different sequences. Notably, 81.3% (13/16) of the shared clones were generated using the soluble immunogen, and identified with both soluble and cell lysate FACS methods. The only three scFv clones that were shared between lymph node and spleen were generated using the cells/DNA immunization method, suggesting that cells/DNA induces a more systemic antigen response than soluble immunization.

**Figure 4:**
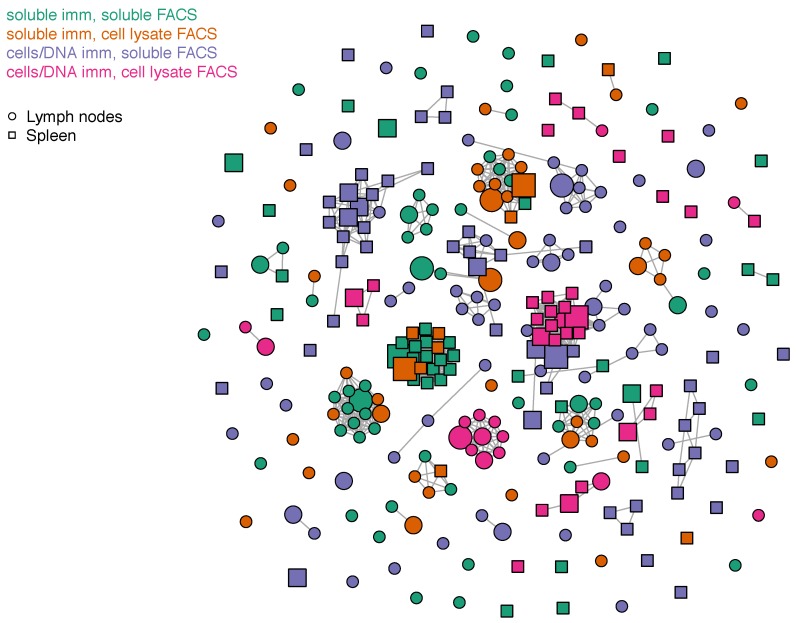
Clonal cluster plot of anti-OX40 clones with frequencies higher than 0.1% in the post-sorted populations. scFv isolated from lymph nodes and spleen are indicated with circles and squares, respectively. scFv isolated from soluble immunization and sorted with either soluble OX40 or cell lysate expressing OX40 are colored in green and orange, respectively. scFv isolated from cell and DNA immunization and sorted with either soluble OX40 or cell lysate expressing OX40 are colored in purple and magenta, respectively. Antibody clones are represented by “nodes” in the plots. The size of the nodes corresponds to the frequency of the antibody clone in the FACS-sorted population: small (<2% frequency), medium (2–12% frequency), and large (>12% frequency). An “edge” (a line linking nodes) was drawn between any sequences with ≤9 amino acid differences in the concatenated scFvs.

A clonal cluster plot of full-length IgH and IgK sequences from scFv binder clones highlights similarities and differences among scFv binder sequences (Figure 4). An “edge” (a line linking nodes) was drawn between any sequences with ≤9 amino acid differences in the concatenated CDR3s. However, most clonal clusters comprised only a single clone, i.e., no related sequences were detected. Only 14 clonal clusters were comprised of five or more scFv clones (putative clonal lineages). Of those, 100% (14/14) comprised clones derived from only the soluble immunogen (using either FACS method) or only cells/DNA immunogen (using either FACS method). In general, sequence analysis suggests that each immunization method, FACS method, and tissue produces mostly unique clones. However, where there is overlap, FACS with different methods is more likely to generate similar clones than different tissues, and different immunization methods are least likely to generate similar clones.

We did not find any significant differences in the sequence characteristics of the most common (≥0.1% frequency) scFv clones between each post-FACS library. Sequence identity to germline (%ID) was high across all methods, averaging 98.4% for IgKV and 97.6% for IgHV. This suggests low levels of affinity maturation in vivo. Variable (V)-gene diversity was low across all methods (Supplementary Figures S9 and S10), for example, 172/268 (64.2%) of clones were some allele of IgHV1, and 72.4% of clones were some allele of IgKV1. In general, scFv clone binders previously identified in Balb/c, SJL, and Medarex HuMAb mice [9,10] showed similarly high levels of germline %ID and similarly low levels of V-gene diversity. Though each immunization and panning method yielded distinct clones (Figure 4), there was a strong bias toward a limited variety of V-Joining (J) combinations, with between-method Pearson correlation coefficients ranging from 0.59 to 0.81 (Supplementary Figure S11), all of which were significant (*p* < 0.001). We postulate that such similarities arise due to the limited V-J diversity in the pre-sort repertoires (Supplementary Figures S9 and S10), though we cannot rule out the possibility that OX40 binding is more likely given particular V-J pairs.

### 3.5. Functional Characteristics of Monoclonal Antibody Binders

Next, to investigate the therapeutic potential of scFv binders, we synthesized forty-one putative binder scFv as full-length mAbs (Table 1), using methods described elsewhere [10]. We chose at least the top two (range: 2–7 scFv) most common scFv enriched in each tissue, from each immunization and FACS method combination, along with several other strong and weak enriched scFv. First, we used a FACS assay to assess the ability of each mAb to bind OX40 recombinantly expressed on cell surfaces. In total, 39% (16/41) of the mAbs bound cell surface antigen, for a 61% false positive rate (Supplementary Figure S5; Supplementary Table S10). Notably, immunizing with soluble OX40 antigen followed by sorting with lysate from OX40-expressing cells yielded a 0% false positive rate, i.e., all mAbs identified with this method bound cell surface OX40. The other methods yielded significantly higher false positive rates (*z*-test for proportions, *p* < 0.01), ranging from 35% to 67%. We also observed two distinct positive binder peaks in the histograms for 68.8% of cell surface binding scFv (11/16), for unknown reasons.

We then tested the sixteen cell-surface binding mAbs for in vitro activation in a cellular assay. The average EC50 was 0.59 μg/μL, with a range from 0.024 to 4.3 μg/μL. Cell surface binding was a good predictor of in vitro agonism, with 87.5% (14/16) of cell surface binders demonstrating agonism (Table 1; Supplementary Figure S7; Supplementary Table S10), for a 77% (27/41) false positive rate overall. Again, immunizing with soluble OX40 antigen followed by sorting with OX40-embedded cell lysate yielded the lowest false positive rate, at 9.1% (1/11). The other methods yielded significantly higher false positive rates (*z*-test for proportions, *p* < 0.01), ranging from 42.1% to 92.9%. The number of peaks in the cell surface binding assay was associated with the strength of agonism: the 1-peak mAbs have an average EC50 of 2.1 (with 2 mAbs not showing any agonist activity), whereas the 2-peak mAbs have an average EC50 of 0.17 (with all mAbs having agonist activity). Note that the positive control benchmark (pogalizumab) is a 2-peak binder with a strong EC50 (0.039 μg/μL). We speculate that mAbs in the 2-peak group comprise a different epitope bin than the mAbs in the 1-peak group. The two mAbs that failed to agonize (tOX40.21 and tOX40.42) also showed the weakest fluorescence shift in the flow cytometry cell surface binding experiments (Supplementary Figure S5). We did not observe significant differences among the protocols in in vitro agonism EC50 (*p* > 0.01, *t*-test).

We also tested the cell surface binding mAbs for affinity using Octet. Of the mAbs that bound cell surface antigen, 93.8% (15/16) also bound soluble antigen (Table 1; Supplementary Figure S6; Supplementary Table S10). The average K_D_ was 41.1 nM, with a range from 2.7 to 184 nM. The pogalizumab positive control yielded a K_D_ of 1.9 nM. One of the antibodies that bound cell surface antigen (tOX40.42) failed to bind antigen by Octet and also failed in vitro agonism. This mAb was discovered using the cells/DNA immunization and soluble antigen sorting method. Another mAb (tOX40.21) did not agonize OX40 in vitro but did bind soluble antigen (K_D_ = 7 nM). This antibody was among the weakest binders in the cell surface flow cytometry assay and was discovered using the soluble antigen immunization with both the soluble sort and cell lysate sort methods. We speculate that this mAb binds non-specifically, resulting in high affinity but weak agonism and cell surface binding. We did not observe significant differences among the protocols in K_D_ (*p* > 0.01, *t*-test).

## 4. Discussion

In this study, we adapted previously published methods [8,9,10] to test whether different immunization methods (cells/DNA versus soluble antigen) and different selection methods (cell lysate versus soluble antigen) yielded mAbs with higher potential as therapeutic OX40 agonists. Though all methods successfully identified anti-OX40 mAbs, using cell lysate for selection generally yielded mAbs that were more likely to bind to cell surface antigen and activate OX40 in cellular assays. We speculate that cell lysate contained OX40 trimers, whereas soluble antigen comprised OX40 monomers, perhaps leading to the identification of more physiologically relevant binders. Using massively parallel microfluidics and deep sequencing allowed us to rigorously characterize mouse responses to different types of immunogens. The large scFv repertoires generated from the animals also facilitated robust testing of FACS methods. Other methods, such as hybridomas, would have required significantly more effort to generate such a comprehensive data set.

In this study, immunization with cells/DNA was inconsistent and yielded a low proportion of agonist mAbs. In future experiments, we could establish a titer cutoff and only make yeast libraries from animals with titers exceeding that cutoff. Still, there are many ways that cells/DNA immunization could be optimized in the future. For example, we could test different concentrations of cells in the mouse immunizations, different adjuvants, different DNA vectors, or alternate cell lines. We could also ensure high levels of cell surface antigen expression by using FACS to isolate populations of cells with the highest antigen expression, as described elsewhere [22]. A more aggressive cells/DNA immunization schedule could increase titer and reproducibility, for example, through daily injections of cells for the first few days of the immunization protocol [22], biweekly immunizations with cells for ten weeks [23], or biweekly immunizations with DNA for eight weeks [24].

Though the cell lysate sorting method yielded the highest proportion of agonist mAbs in this study, there are many opportunities for further improvement. We tested several different cell lysate concentrations, but a more rigorous optimization would require a more thorough analysis of the impact of lysate concentration on FACS sensitivity and specificity. We only tested the cell lysate sorting methods with OX40, whereas other targets may yield different results. For example, certain targets may unfold in our lysis buffer, yielding antibodies less likely to bind to a properly folded protein target. Additionally, we might find that peptide tags other than FLAG (for example, His tag) might yield better results, or that C-terminal tags are preferential for certain targets. Finally, further work might compare screening yeast scFv libraries with cell lysate versus screening phage scFv display libraries against cells affixed to plates.

To our knowledge, no other group has published in-depth studies of the antibody repertoire response of Trianni humanized mice to immunogens. Our work yielded a low diversity of light chain V-genes, for example, >70% of scFv binder clones were IgKV1. This level of light chain Ig diversity after immunization and FACS selection is similar to results obtained in wild type Balb/c and SJL mice [9] and humanized Medarex HuMAb mice [10]. Additionally, the V sequences of scFv binders from both libraries were ~98% identical to germline V sequences, suggesting little if any affinity maturation in vivo. Prior work on repertoires of mice administered various immunogens found only 2–5 amino acid substitutions per V-gene [9,10,25,26,27]. Future work should investigate whether Trianni mice generate similar responses with other immunogens.

Our methods open up exciting directions for mAb discovery and development. For example, we could use cell lysates to select for mAbs that bind to a specific epitope, or do not bind to a specific epitope. In one scenario, cells could be engineered that express OX40 protein with mutations in the amino acids required for binding OX40L. Then, scFv libraries could be sorted using the mutated OX40, perhaps identifying antibodies that bind outside the OX40:OX40L binding domain. Another intriguing approach would be to immunize mice with tumor cells, and then pan for scFv that bind to lysates from tumors but not to lysates from normal tissue. This approach could be used to find mAbs directed against novel tumor-specific targets.

## 5. Patents

The OX40 full-length antibody sequences and the OX40-enriched yeast scFv libraries described in this article are patent-pending subject matter in USPTO provisional patent application number 62/788687, priority date 4 January 2019.

## Figures and Tables

**Table 1:** Functional characteristics of 41 scFv binders converted into full-length IgG1 mAbs.

mAb ID	Enriched? Soluble Immunized, Soluble Sorted	Enriched? Soluble Immunized, Lysate Sorted	Enriched? Cells/DNA Immunized, Soluble Sorted	Enriched? Cells/DNA Immunized, Lysate Sorted	Binds Cells?	Promega In Vitro Assay EC50 (ug/mL) (Agonist)	KD (nM) [Octet, Global Fit]
tOX40.2	Yes	No	No	No	No	not tested	not tested
tOX40.4	Yes	Yes	No	No	Yes (2 peaks)	0.044	9
tOX40.15	Yes	No	No	No	Yes (1 peak)	1.276	6
tOX40.19	Yes	Yes	No	No	Yes (1 peak)	0.811	7.7
tOX40.20	Yes	No	No	No	No	not tested	not tested
tOX40.21	Yes	Yes	No	No	Yes (1 peak)	does not agonize	7
tOX40.22	Yes	Yes	No	No	Yes (2 peaks)	0.087	7.3
tOX40.23	Yes	Yes	No	No	Yes (2 peaks)	0.419	5.2
tOX40.24	Yes	Yes	No	No	Yes (2 peaks)	0.024	22.9
tOX40.28	Yes	No	No	No	No	not tested	not tested
tOX40.31	Yes	Yes	No	No	Yes (2 peaks)	0.043	22.4
tOX40.33	Yes	No	No	No	No	not tested	not tested
tOX40.34	Yes	No	No	No	No	not tested	not tested
tOX40.35	Yes	No	No	No	Yes (1 peak)	4.316	2.7
tOX40.36	Yes	No	No	No	No	not tested	not tested
tOX40.37	Yes	Yes	No	No	Yes (2 peaks)	0.195	12.5
tOX40.38	Yes	No	No	No	No	not tested	not tested
tOX40.39	Yes	Yes	No	No	Yes (2 peaks)	0.199	90
tOX40.40	Yes	Yes	No	No	Yes (2 peaks)	0.426	151
tOX40.41	No	Yes	No	No	Yes (2 peaks)	0.068	58.4
tOX40.42	No	No	Yes	No	Yes (1 peak)	does not agonize	no binding
tOX40.43	No	No	Yes	No	No	not tested	not tested
tOX40.44	No	No	Yes	No	No	not tested	not tested
tOX40.45	No	No	Yes	No	No	not tested	not tested
tOX40.46	No	No	Yes	No	No	not tested	not tested
tOX40.47	No	No	Yes	No	No	not tested	not tested
tOX40.48	No	No	Yes	No	No	not tested	not tested
tOX40.49	No	No	Yes	No	No	not tested	not tested
tOX40.50	No	No	Yes	Yes	Yes (2 peaks)	0.091	29.8
tOX40.51	No	No	No	Yes	No	not tested	not tested
tOX40.52	No	No	No	Yes	No	not tested	not tested
tOX40.54	No	No	No	Yes	Yes (2 peaks)	0.321	184
tOX40.55	No	No	No	Yes	No	not tested	not tested
tOX40.56	No	No	Yes	No	No	not tested	not tested
tOX40.57	No	No	Yes	No	No	not tested	not tested
tOX40.58	No	No	Yes	No	No	not tested	not tested
tOX40.59	No	No	Yes	No	No	not tested	not tested
tOX40.60	No	No	Yes	No	No	not tested	not tested
tOX40.61	No	No	No	Yes	No	not tested	not tested
tOX40.62	No	No	No	Yes	No	not tested	not tested
tOX40.63	No	No	No	Yes	No	not tested	not tested

## Supplementary Materials

The following are available online at http://www.mdpi.com/2073-4468/8/1/17/s1, Figure S1: Serum titers for five mice immunized with soluble OX40 extracellular domain, as measured by ELISA, Figure S2: Vector map for DNA construct used to stably transfect FLAG-tagged OX40 antigen in CHO cells, Figure S3: Serum titers for three mice immunized with soluble OX40 extracellular domain, as measured by binding to cells expressing recombinant OX40, followed by flow cytometry, Figure S4: A fourth round of panning for experiments that had generated a relatively low proportion of scFv binders after three rounds of panning, Figure S5: Assessment of mAb binding to CHO cell surface human OX40 antigen or an irrelevant CHO cell surface control, using flow cytometry, Figure S6: Assessment of kinetics of mAb binding to soluble OX40 antigen, using Octet, Figure S7: Assessment of OX40 agonism by candidate mAbs, using a Promega in vitro cell assay, Figure S8: Panning for OX40 scFv binders using four different cell lysate concentrations, Figure S9: Heatmap showing clone count for Ig heavy chain V-J pairings for soluble immunized, cells/DNA immunized, Figure S10: Heatmap showing clone count for Ig light chain V-J pairings for soluble immunized, cells/DNA immunized, soluble antigen FACS, and cell lysate FACS experiments, Figure S11: Scatter plots and Pearson correlation coefficients in V-J clone counts between cells/DNA immunization with cell lysate FACS, cells/DNA immunization with soluble antigen FACS, soluble antigen immunization with cell lysate FACS, and soluble antigen immunization with soluble antigen FACS, Table S1: Counts of input cells, sequence reads obtained, and scFv clones identified for experiments reported in the study, Table S2: sequences, FACS enrichments, and germline alignments for scFv clones identified in lymph node tissue using cells/DNA immunization, cell lysate sorting, Table S3: sequences, FACS enrichments, and germline alignments for scFv clones identified in spleen tissue using cells/DNA immunization, cell lysate sorting, Table S4: sequences, FACS enrichments, and germline alignments for scFv clones identified in lymph node tissue using cells/DNA immunization, soluble OX40 sorting, Table S5: sequences, FACS enrichments, and germline alignments for scFv clones identified in spleen tissue using cells/DNA immunization, soluble OX40 sorting, Table S6: sequences, FACS enrichments, and germline alignments for scFv clones identified in lymph node tissue using soluble OX40 immunization, cell lysate sorting, Table S7: sequences, FACS enrichments, and germline alignments for scFv clones identified in spleen tissue using soluble OX40 immunization, cell lysate sorting, Table S8: sequences, FACS enrichments, and germline alignments for scFv clones identified in lymph node tissue using soluble OX40 immunization, soluble OX40 sorting, Table S9: sequences, FACS enrichments, and germline alignments for scFv clones identified in spleen tissue using soluble OX40 immunization, soluble OX40 sorting, Table S10: CDR3s for full-length monoclonal antibodies generated, corresponding scFv identifiers, cell surface binding data, cell activation assay data, binding kinetics, and corresponding sequence frequencies in pre- and post-FACS scFv repertoires.
