# Supplementary material for: Preferential Identification of Agonistic OX40 Antibodies by Using Cell Lysate to Pan Natively Paired, Humanized Mouse-Derived Yeast Surface Display Libraries
Source: Antibodies (Basel). 2019 Feb 19;8(1):17. doi: 10.3390/antib8010017 (PMC6640694; doi:10.3390/antib8010017)
Supplement: Supplementary file 1 [file antibodies-08-00017-s001.zip › Supplementary Files (OX40)/Supplementary Figures (cell lysate).pptx]

## Slide 1
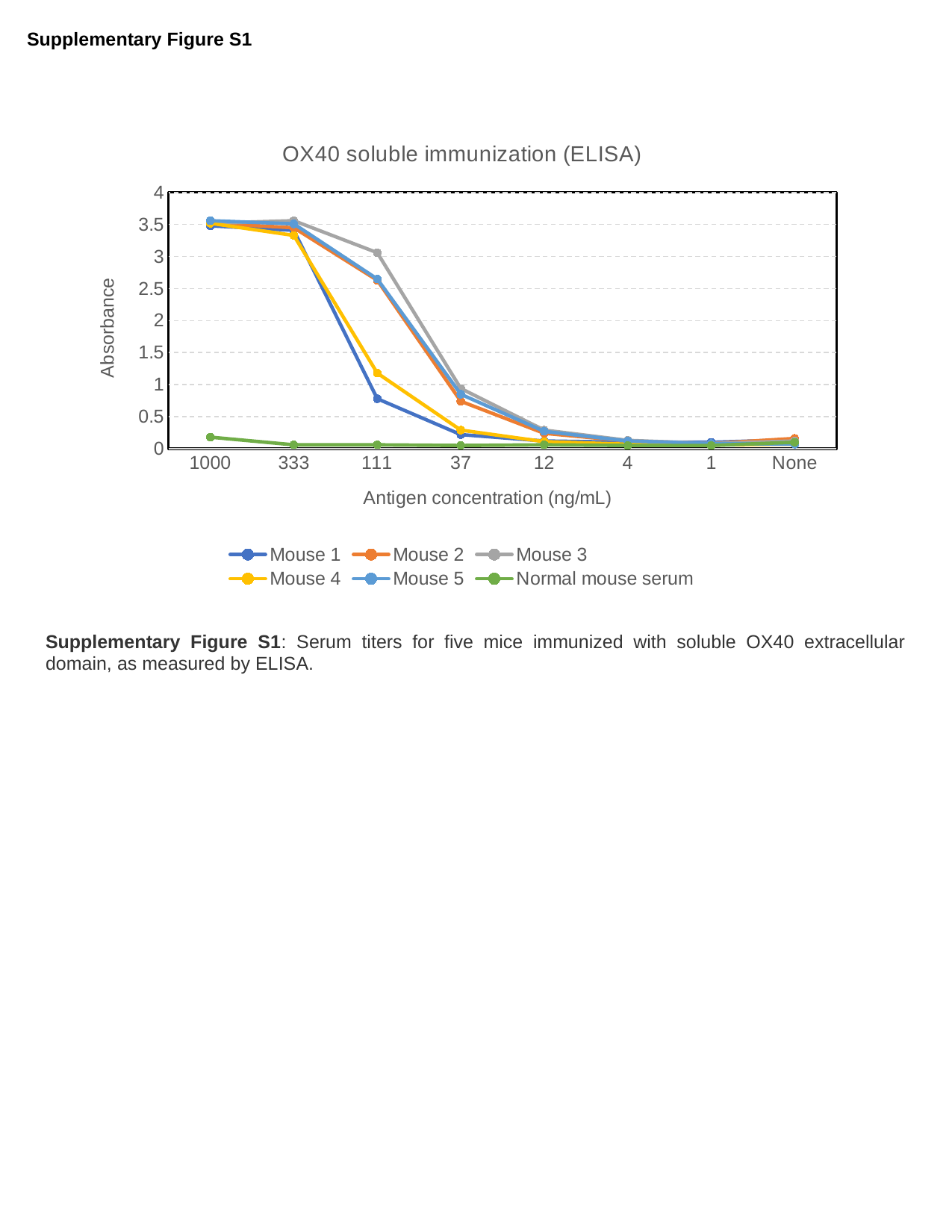

Supplementary Figure S1
### Chart: OX40 soluble immunization (ELISA)
| Category | Mouse 1 | Mouse 2 | Mouse 3 | Mouse 4 | Mouse 5 | Normal mouse serum |
|---|---|---|---|---|---|---|
| 1000 | 3.48 | 3.52 | 3.52 | 3.52 | 3.56 | 0.18 |
| 333 | 3.41 | 3.45 | 3.56 | 3.33 | 3.51 | 0.06 |
| 111 | 0.78 | 2.63 | 3.06 | 1.18 | 2.65 | 0.06 |
| 37 | 0.22 | 0.74 | 0.94 | 0.29 | 0.85 | 0.05 |
| 12 | 0.12 | 0.24 | 0.29 | 0.11 | 0.27 | 0.06 |
| 4 | 0.1 | 0.12 | 0.13 | 0.07 | 0.12 | 0.05 |
| 1 | 0.1 | 0.08 | 0.08 | 0.06 | 0.08 | 0.05 |
| None | 0.14 | 0.16 | 0.12 | 0.07 | 0.07 | 0.1 |Supplementary Figure S1: Serum titers for five mice immunized with soluble OX40 extracellular domain, as measured by ELISA.

## Slide 2
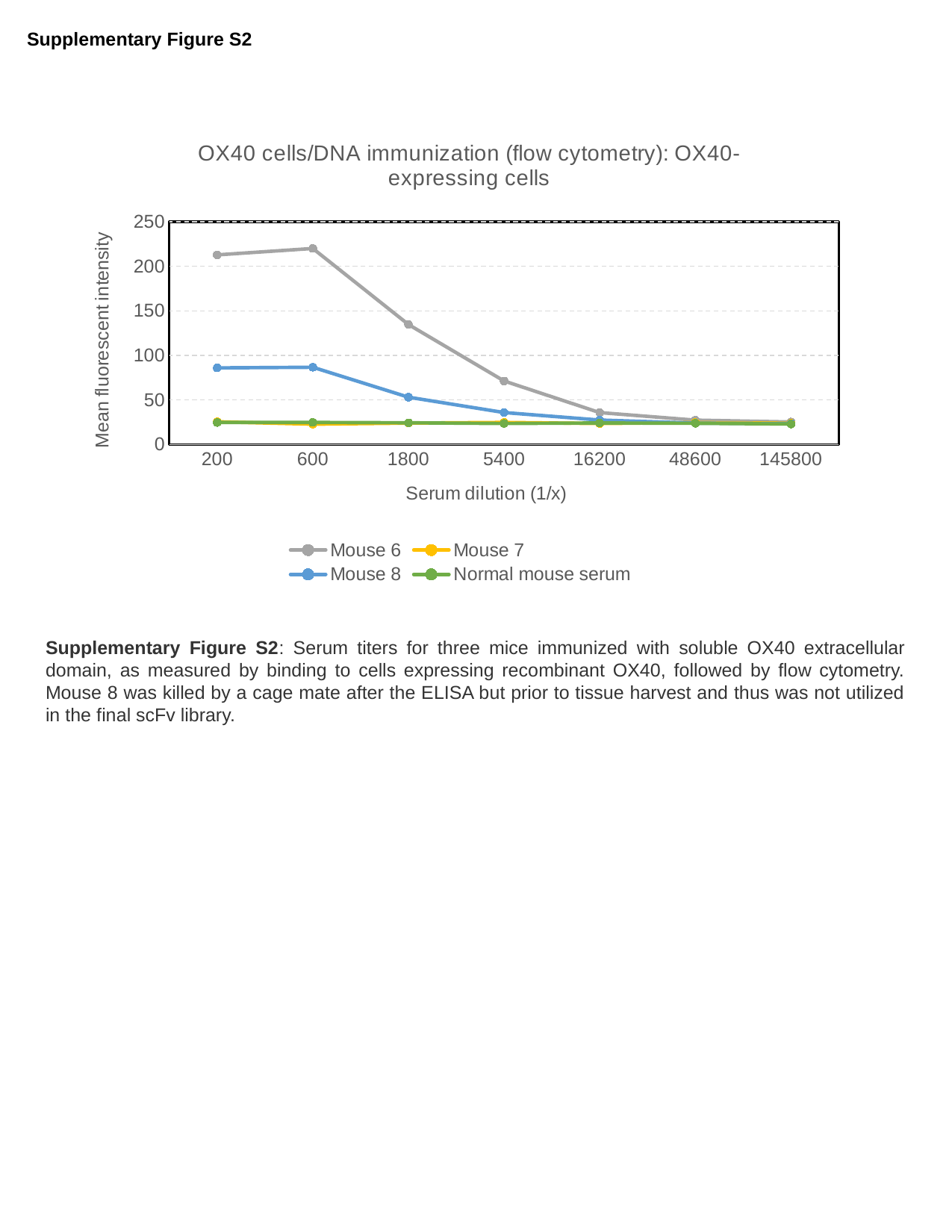

Supplementary Figure S2
### Chart: OX40 cells/DNA immunization (flow cytometry): OX40-expressing cells
| Category | Mouse 6 | Mouse 7 | Mouse 8 | Normal mouse serum |
|---|---|---|---|---|
| 200 | 212.79 | 25.45 | 85.91 | 24.64 |
| 600 | 219.95 | 22.73 | 86.62 | 24.71 |
| 1800 | 134.6 | 23.96 | 53.02 | 24.33 |
| 5400 | 71.2 | 24.72 | 35.79 | 23.54 |
| 16200 | 35.76 | 23.47 | 27.4 | 24.13 |
| 48600 | 27.27 | 24.57 | 23.93 | 23.72 |
| 145800 | 25.21 | 23.93 | 23.36 | 22.91 |Supplementary Figure S2: Serum titers for three mice immunized with soluble OX40 extracellular domain, as measured by binding to cells expressing recombinant OX40, followed by flow cytometry. Mouse 8 was killed by a cage mate after the ELISA but prior to tissue harvest and thus was not utilized in the final scFv library.

## Slide 3
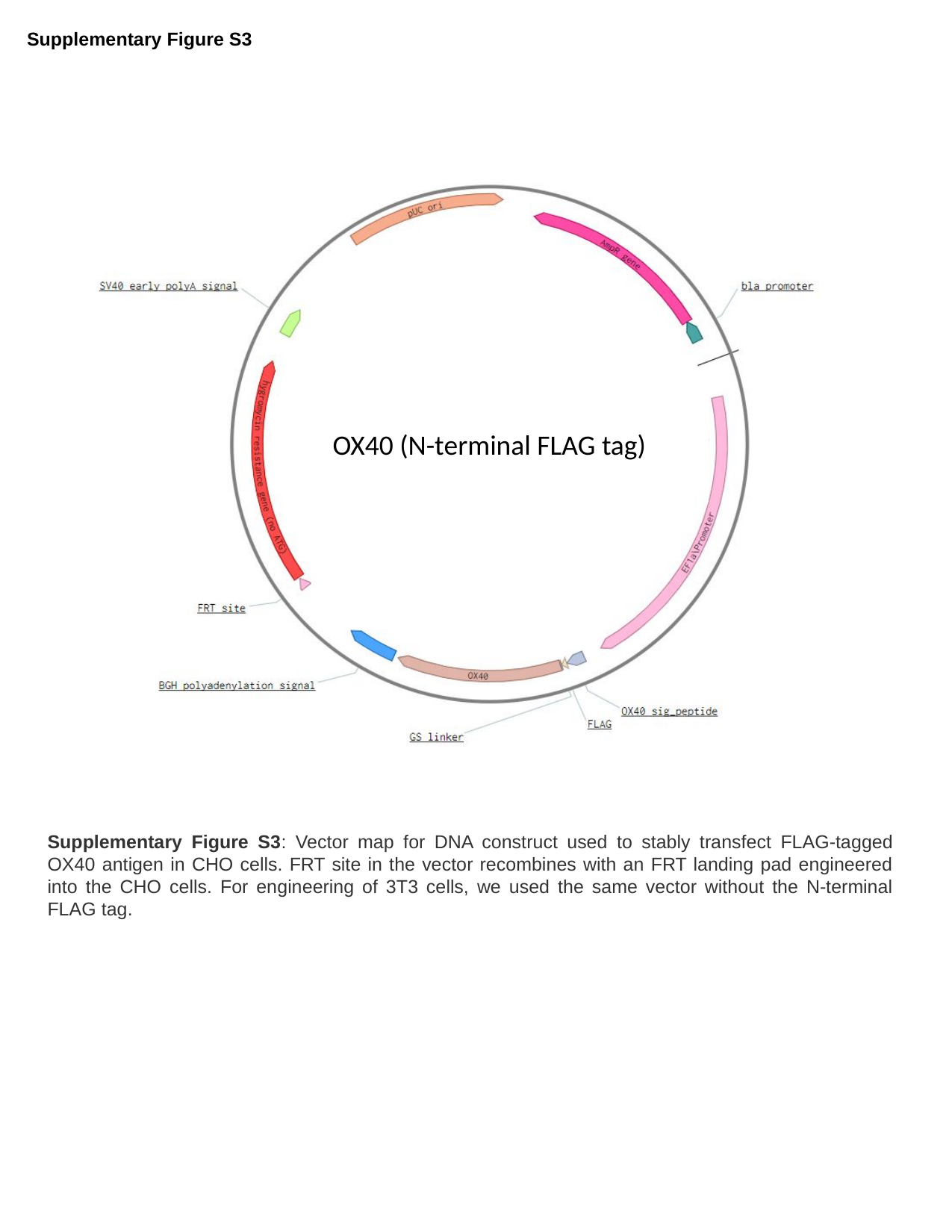

Supplementary Figure S3
OX40 (N-terminal FLAG tag)
Supplementary Figure S3: Vector map for DNA construct used to stably transfect FLAG-tagged OX40 antigen in CHO cells. FRT site in the vector recombines with an FRT landing pad engineered into the CHO cells. For engineering of 3T3 cells, we used the same vector without the N-terminal FLAG tag.

## Slide 4
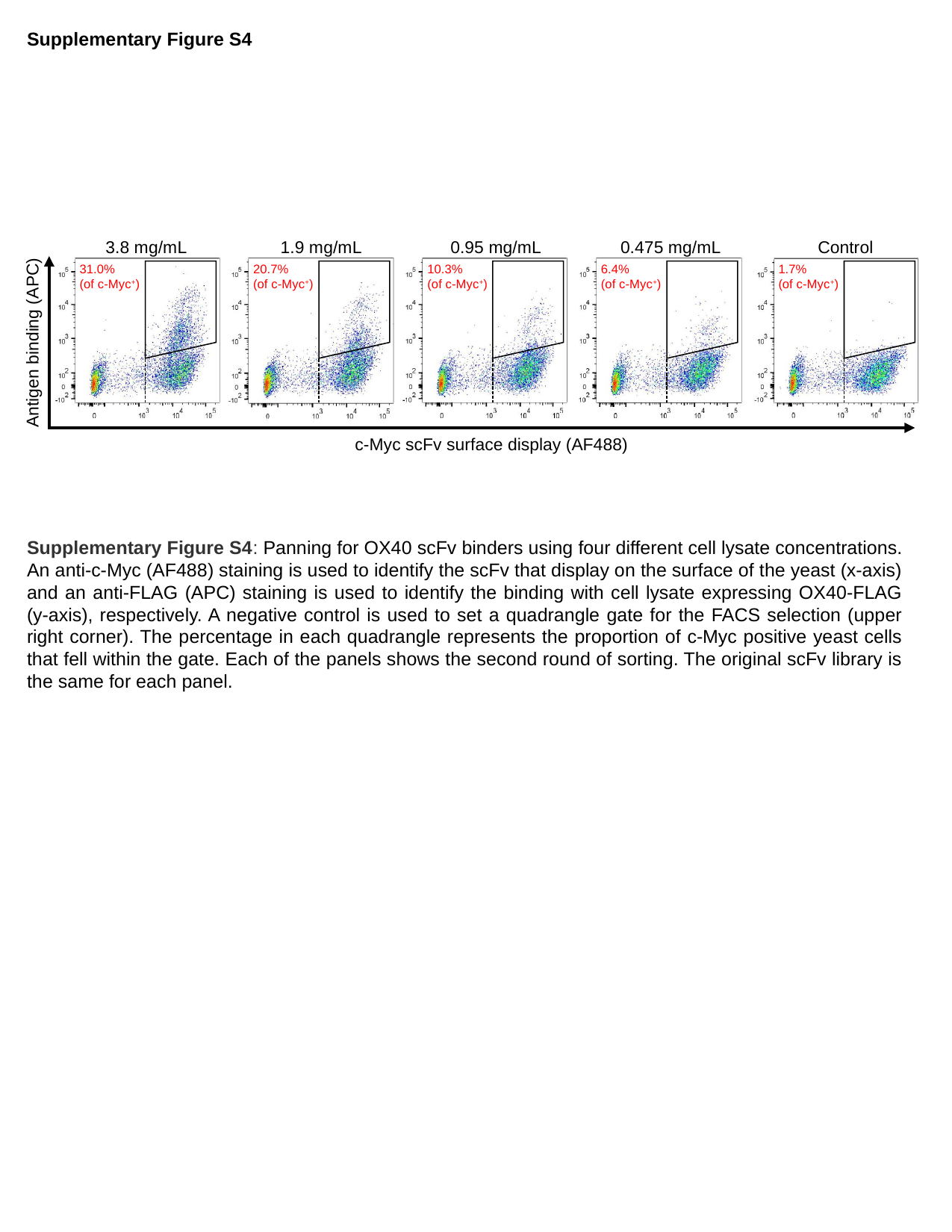

Supplementary Figure S4
3.8 mg/mL
1.9 mg/mL
0.95 mg/mL
0.475 mg/mL
Control
31.0%
(of c-Myc+)
20.7%
(of c-Myc+)
10.3%
(of c-Myc+)
6.4%
(of c-Myc+)
1.7%
(of c-Myc+)
Antigen binding (APC)
c-Myc scFv surface display (AF488)
Supplementary Figure S4: Panning for OX40 scFv binders using four different cell lysate concentrations. An anti-c-Myc (AF488) staining is used to identify the scFv that display on the surface of the yeast (x-axis) and an anti-FLAG (APC) staining is used to identify the binding with cell lysate expressing OX40-FLAG (y-axis), respectively. A negative control is used to set a quadrangle gate for the FACS selection (upper right corner). The percentage in each quadrangle represents the proportion of c-Myc positive yeast cells that fell within the gate. Each of the panels shows the second round of sorting. The original scFv library is the same for each panel.

## Slide 5
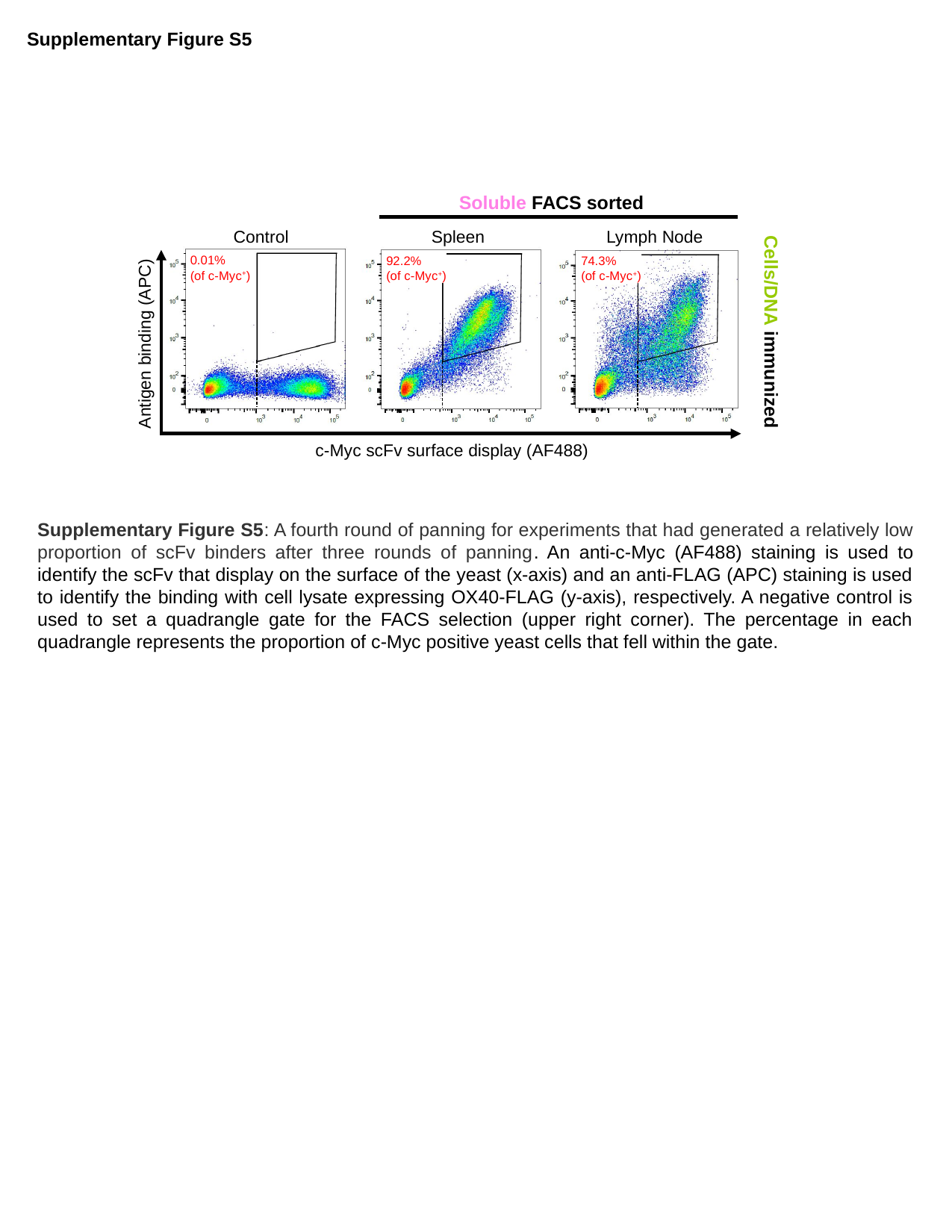

Supplementary Figure S5
Soluble FACS sorted
Control
Spleen
Lymph Node
0.01%
(of c-Myc+)
92.2%
(of c-Myc+)
74.3%
(of c-Myc+)
Cells/DNA immunized
Antigen binding (APC)
c-Myc scFv surface display (AF488)
Supplementary Figure S5: A fourth round of panning for experiments that had generated a relatively low proportion of scFv binders after three rounds of panning. An anti-c-Myc (AF488) staining is used to identify the scFv that display on the surface of the yeast (x-axis) and an anti-FLAG (APC) staining is used to identify the binding with cell lysate expressing OX40-FLAG (y-axis), respectively. A negative control is used to set a quadrangle gate for the FACS selection (upper right corner). The percentage in each quadrangle represents the proportion of c-Myc positive yeast cells that fell within the gate.

## Slide 6
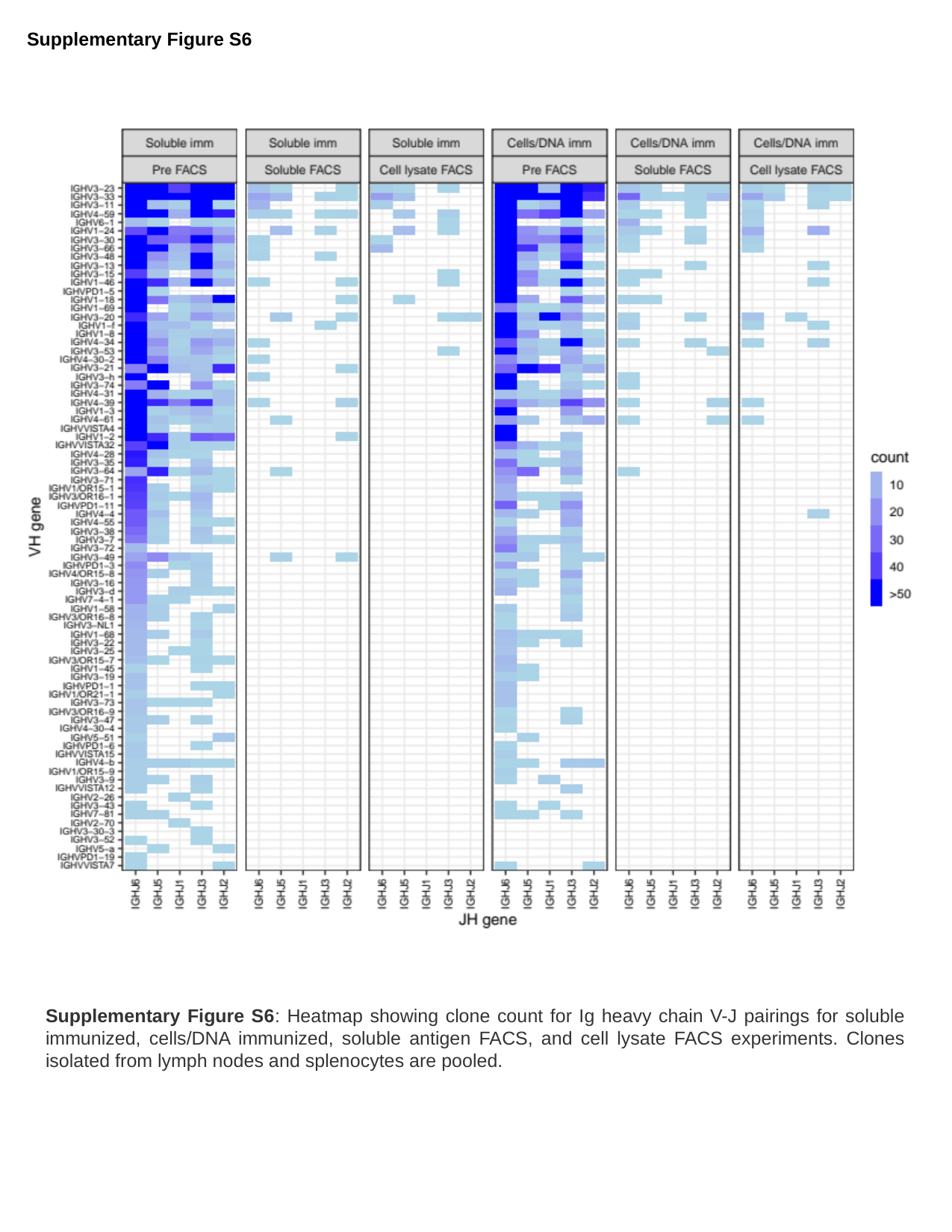

Supplementary Figure S6
Supplementary Figure S6: Heatmap showing clone count for Ig heavy chain V-J pairings for soluble immunized, cells/DNA immunized, soluble antigen FACS, and cell lysate FACS experiments. Clones isolated from lymph nodes and splenocytes are pooled.

## Slide 7
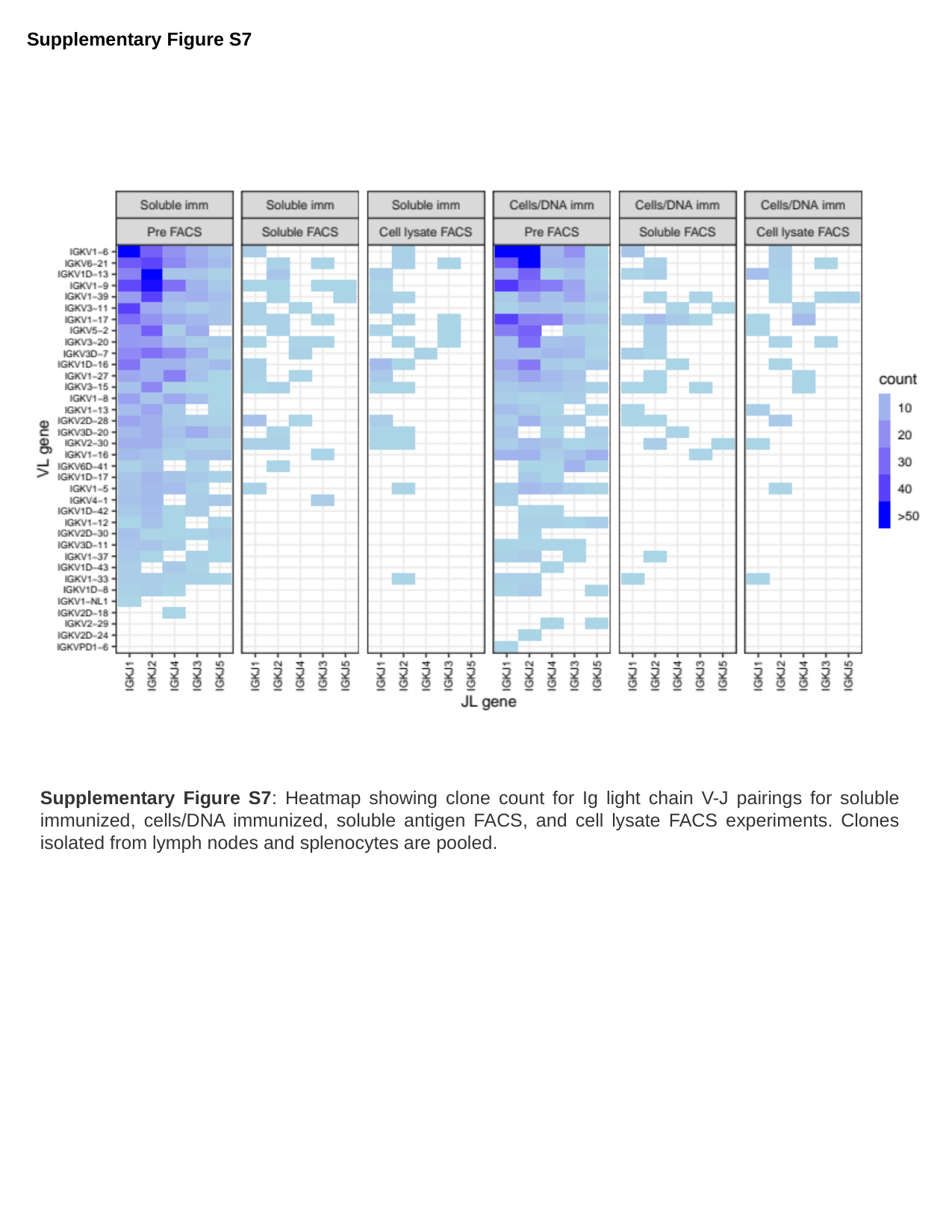

Supplementary Figure S7
Supplementary Figure S7: Heatmap showing clone count for Ig light chain V-J pairings for soluble immunized, cells/DNA immunized, soluble antigen FACS, and cell lysate FACS experiments. Clones isolated from lymph nodes and splenocytes are pooled.

## Slide 8
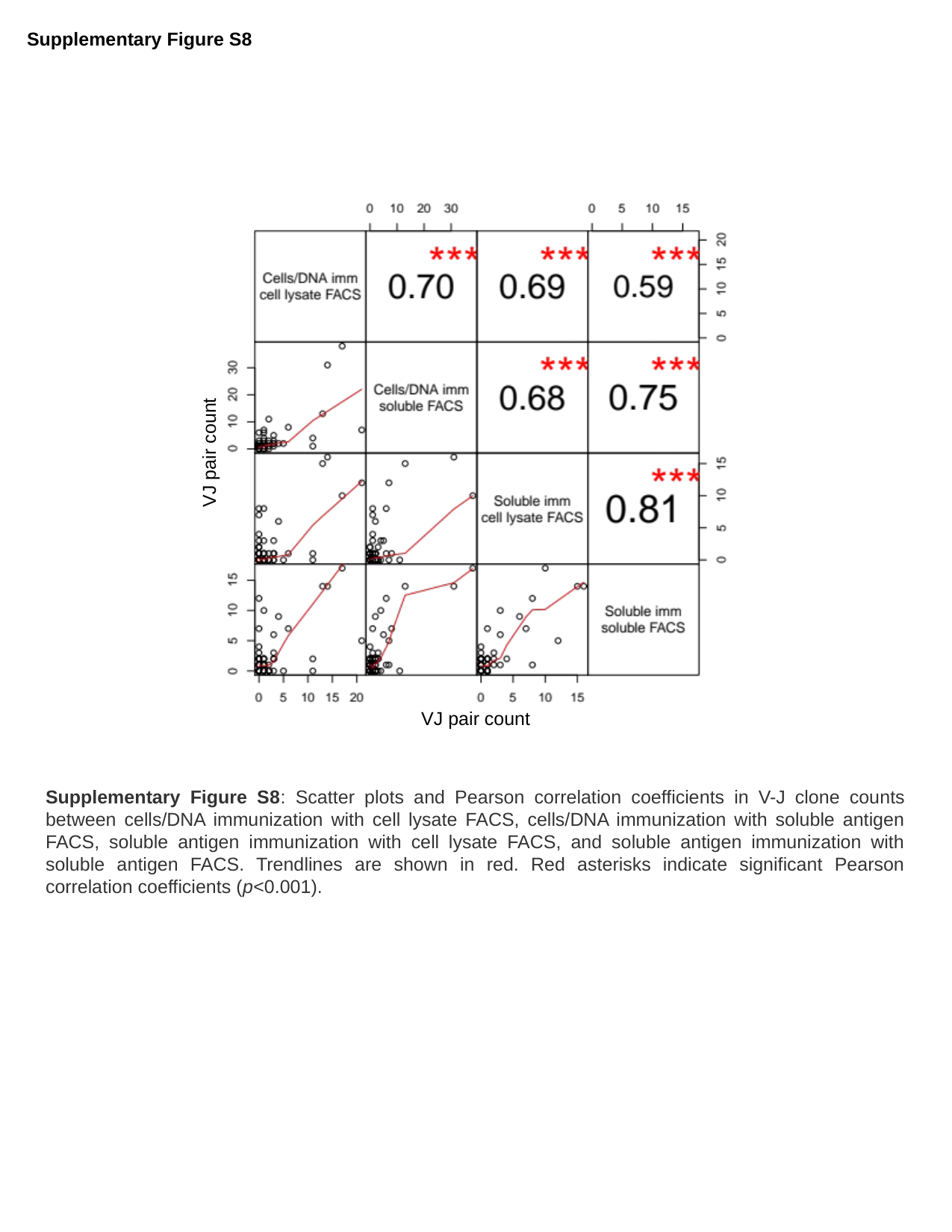

Supplementary Figure S8
VJ pair count
VJ pair count
Supplementary Figure S8: Scatter plots and Pearson correlation coefficients in V-J clone counts between cells/DNA immunization with cell lysate FACS, cells/DNA immunization with soluble antigen FACS, soluble antigen immunization with cell lysate FACS, and soluble antigen immunization with soluble antigen FACS. Trendlines are shown in red. Red asterisks indicate significant Pearson correlation coefficients (p<0.001).

## Slide 9
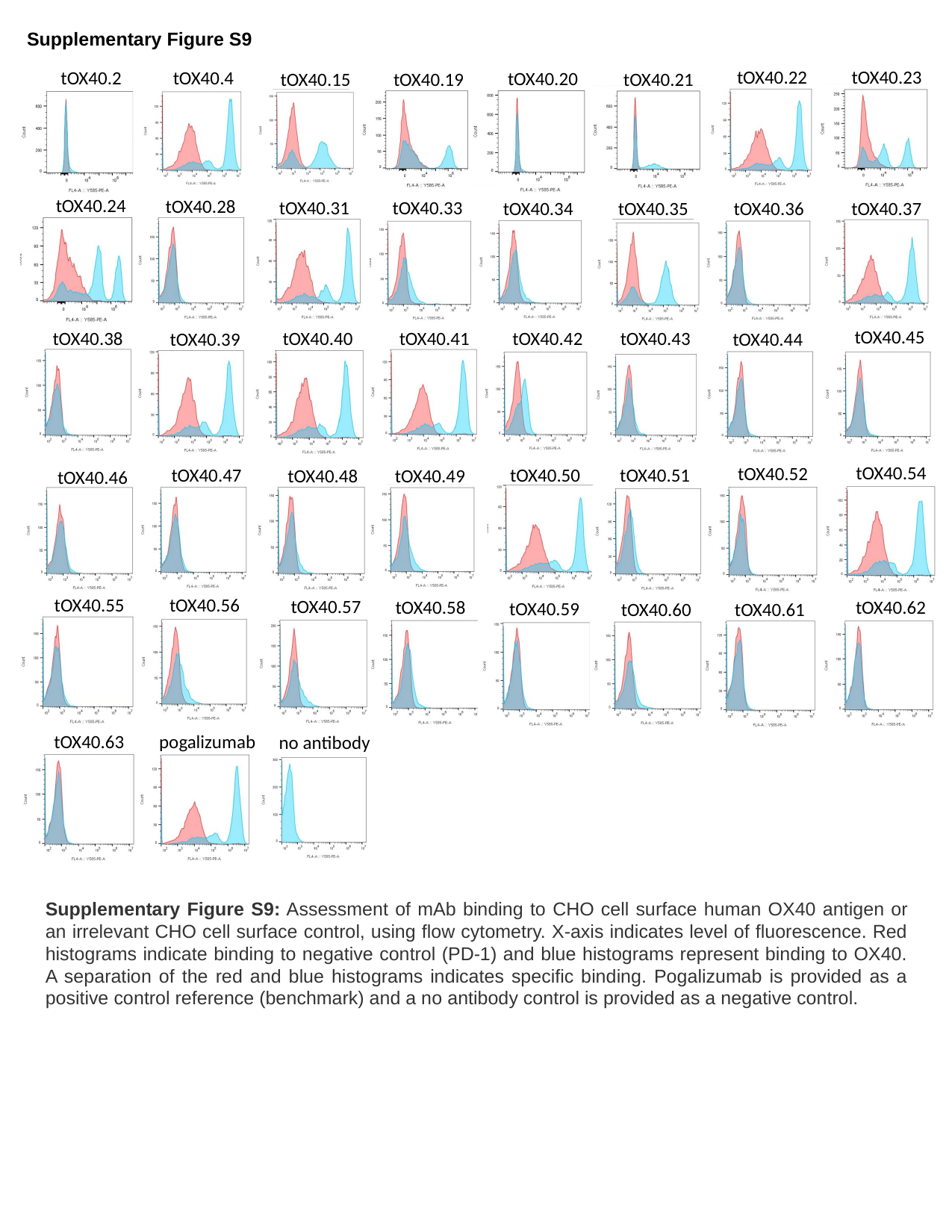

Supplementary Figure S9
tOX40.23
tOX40.22
tOX40.2
tOX40.4
tOX40.20
tOX40.21
tOX40.15
tOX40.19
tOX40.24
tOX40.28
tOX40.33
tOX40.31
tOX40.37
tOX40.36
tOX40.34
tOX40.35
tOX40.45
tOX40.41
tOX40.40
tOX40.38
tOX40.42
tOX40.43
tOX40.44
tOX40.39
tOX40.54
tOX40.52
tOX40.51
tOX40.50
tOX40.47
tOX40.49
tOX40.48
tOX40.46
tOX40.56
tOX40.55
tOX40.57
tOX40.62
tOX40.58
tOX40.59
tOX40.61
tOX40.60
pogalizumab
tOX40.63
no antibody
Supplementary Figure S9: Assessment of mAb binding to CHO cell surface human OX40 antigen or an irrelevant CHO cell surface control, using flow cytometry. X-axis indicates level of fluorescence. Red histograms indicate binding to negative control (PD-1) and blue histograms represent binding to OX40. A separation of the red and blue histograms indicates specific binding. Pogalizumab is provided as a positive control reference (benchmark) and a no antibody control is provided as a negative control.

## Slide 10
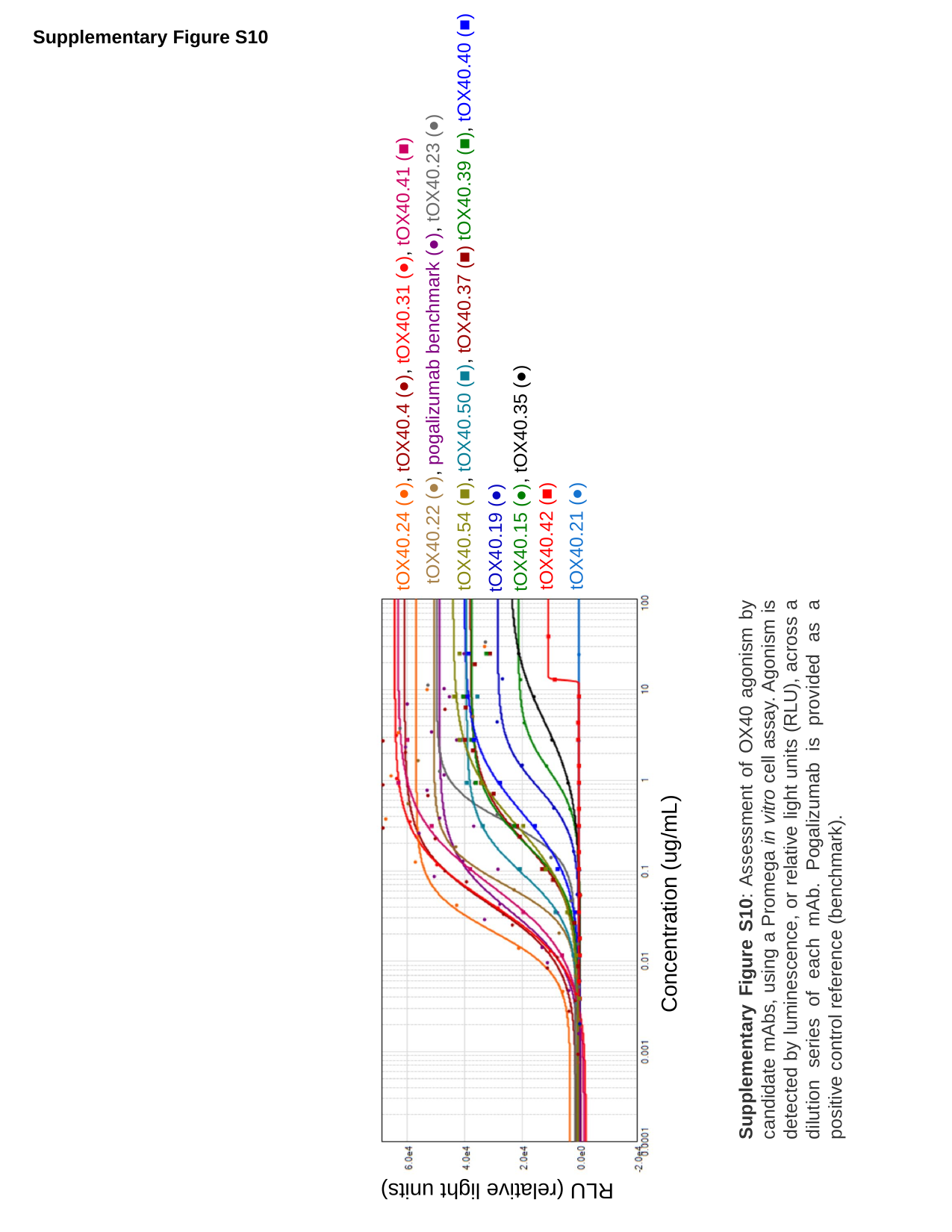

Supplementary Figure S10
tOX40.54 (■), tOX40.50 (■), tOX40.37 (■) tOX40.39 (■), tOX40.40 (■)
tOX40.22 (●), pogalizumab benchmark (●), tOX40.23 (●)
tOX40.24 (●), tOX40.4 (●), tOX40.31 (●), tOX40.41 (■)
tOX40.15 (●), tOX40.35 (●)
tOX40.21 (●)
tOX40.42 (■)
tOX40.19 (●)
Supplementary Figure S10: Assessment of OX40 agonism by candidate mAbs, using a Promega in vitro cell assay. Agonism is detected by luminescence, or relative light units (RLU), across a dilution series of each mAb. Pogalizumab is provided as a positive control reference (benchmark).
Concentration (ug/mL)
RLU (relative light units)

## Slide 11
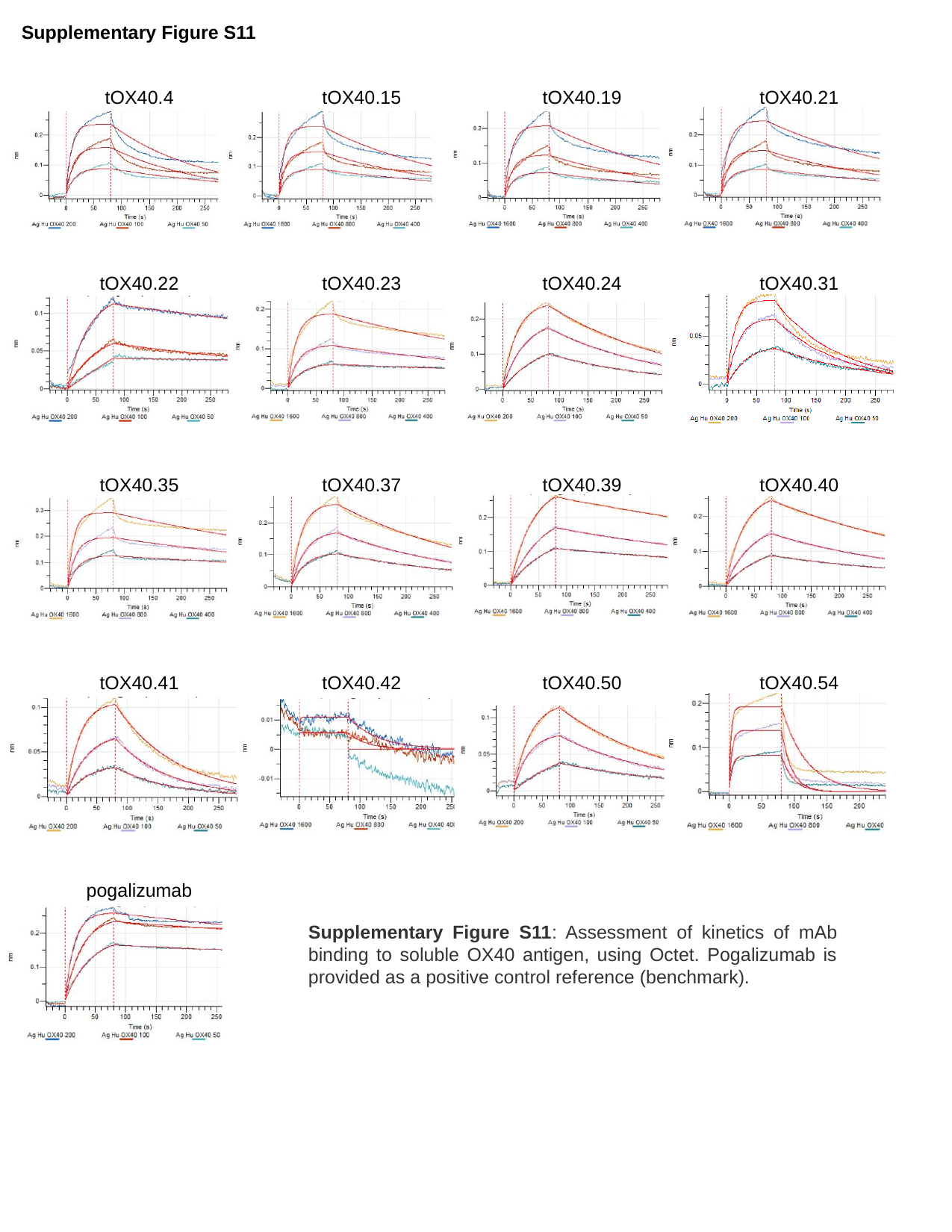

Supplementary Figure S11
tOX40.4
tOX40.15
tOX40.19
tOX40.21
tOX40.22
tOX40.23
tOX40.24
tOX40.31
tOX40.35
tOX40.37
tOX40.39
tOX40.40
tOX40.41
tOX40.42
tOX40.50
tOX40.54
pogalizumab
Supplementary Figure S11: Assessment of kinetics of mAb binding to soluble OX40 antigen, using Octet. Pogalizumab is provided as a positive control reference (benchmark).
